# The minimum area requirements (MAR) for giant panda: an empirical study

**DOI:** 10.1038/srep37715

**Published:** 2016-12-08

**Authors:** Jing Qing, Zhisong Yang, Ke He, Zejun Zhang, Xiaodong Gu, Xuyu Yang, Wen Zhang, Biao Yang, Dunwu Qi, Qiang Dai

**Affiliations:** 1 Key Laboratory of Southwest China Wildlife Resources Conservation (Ministry of Education), China West Normal University, Nanchong, 637002, China; 2 Chengdu Institute of Biology, Chinese Academy of Sciences, Chengdu, 610041, China; 3 Institute of Rare Animals and Plants, China West Normal University, Nanchong, 637002, China; 4 Sichuan Station of Wild life survey and Management, Chengdu, 610082, China; 5 Sichuan Provincial Institute of Forestry Survey and Planning, Chengdu, 610082, China; 6 Conservation International, Chengdu, 610064, China; 7 Chengdu Research Base of Giant Panda Breeding, Sichuan Key Laboratory of Conservation Biology for Endangered Wildlife, Chengdu, 610086, China

## Abstract

Habitat fragmentation can reduce population viability, especially for area-sensitive species. The Minimum Area Requirements (MAR) of a population is the area required for the population’s long-term persistence. In this study, the response of occupancy probability of giant pandas against habitat patch size was studied in five of the six mountain ranges inhabited by giant panda, which cover over 78% of the global distribution of giant panda habitat. The probability of giant panda occurrence was positively associated with habitat patch area, and the observed increase in occupancy probability with patch size was higher than that due to passive sampling alone. These results suggest that the giant panda is an area-sensitive species. The MAR for giant panda was estimated to be 114.7 km^2^ based on analysis of its occupancy probability. Giant panda habitats appear more fragmented in the three southern mountain ranges, while they are large and more continuous in the other two. Establishing corridors among habitat patches can mitigate habitat fragmentation, but expanding habitat patch sizes is necessary in mountain ranges where fragmentation is most intensive.

Habitat fragmentation poses serious threats to many species and to global biodiversity^1^,^2^,^3^, including reductions in population viability^4^,^5^,^6^. Animal species absent or rare in small habitat patches are called ‘area-sensitive’ species^7^,^8^,^9^. Researchers have documented the ‘area-sensitive’ species in almost all taxonomic groups of wildlife, including insects^10^,^11^, fish^12^, reptiles and amphibians^13^, mammals^5^,^14^ and birds^15^,^16^.

The most effective way to conserve area-sensitive species is to maintain habitat patches large enough for the persistence of local populations. Therefore, estimating habitat patch area requirements^17^,^18^ is an essential component of conservation plans. The Minimum Area Requirements (MAR) of a species is defined as the amount of space (suitable habitat) that is required for the long-term persistence of a population^19^. Recently, an increasing number of studies have focused on MAR for protecting animals and highlighted the importance of this measure for conservation decisions^12^,^20^,^21^,^22^. Despite the conservation significance of MAR, empirical studies estimating it remain limited^19^.

The giant panda (*Ailuropoda melanoleuca*) is regarded as one of the most imperiled mammals in the world^23^. Populations of giant panda originally extended throughout most of southern and eastern China, northern Myanmar, and northern Vietnam^24^. Giant panda habitat is dramatically degrading due to increasing human activities and natural catastrophes^25^,^26^,^27^,^28^. Current remnant populations are restricted to six separate regions scattered throughout rugged mountain ranges at the eastern edge of the Tibetan Plateau^29^,^30^. Within those regions, the habitats are highly fragmented^31^,^32^,^33^,^34^, which drives further decreases in total giant panda numbers^35^.

Many studies have been conducted on the habitat of giant panda, and most of them focused on habitat selection^36^,^37^,^38^, habitat quality assessment^39^,^40^,^41^ and the impacts of human activity^27^,^42^,^43^. However, little work has been done on the effects of habitat patch size on giant panda populations. Giant pandas are generally regarded as an area-sensitive species in previous studies^44^, but empirical evidence is still lacking. This study seeks to understand how giant panda population presence depends on patch size and to test whether the giant panda are area-sensitive. We use extensive empirical datasets on giant panda presence and environmental variables to evaluate the MAR for giant panda in five mountain ranges, covering more than 78.7% of giant panda habitat and supporting more than 74.4% of giant pandas remaining in the wild^45^. We also examine the characteristics of remnant habitat patches in these mountain ranges, which collectively comprise the most important giant panda habitat in the world.

## Results

Using the giant panda presence records and environmental variables, we estimated habitat suitability index (HSI). The area under the curve (AUC) value of 0.843 indicated that the Maximum Entropy (MaxEnt) model of giant panda presence had a good discriminatory ability, while the true skill statistic (TSS) score of 0.772 indicated an excellent discriminatory ability of MaxEnt. By the cut-off value that maximized the TSS score, we identified 5569 habitat patches with a total area of 15475.53 km^2^. The habitat patches clustered in five mountain ranges: Minshan Mountains (MS Mountains), Qionglaishan Mountains (QLS Mountains), Daxiangling Mountains (DXL Mountains), Xiaoxiangling Mountains (XXL Mountains) and Liangshan Mountains (LS Mountains) (Table 1).

**Figure 1:**
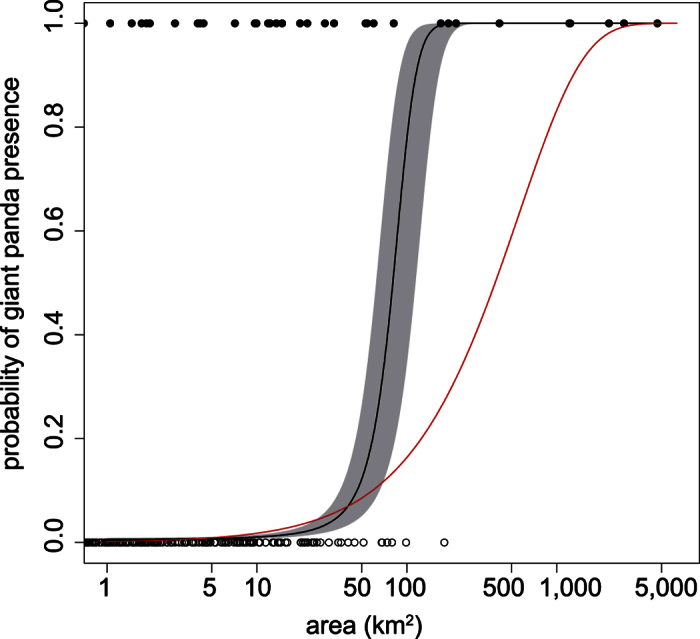
Relationship between the probability of giant panda presence and habitat size. Open circles are patches without indicators of giant panda presence and filled circles are patches where giant pandas were present. The black line is the logistic regression fit, and the grey region shows 95% confidence intervals. The red line represents the null response curve derived from pure passive sampling.

A logistic regression model for the response of occupancy probability to patch area showed a positive association between occupancy probability and habitat area (*P* < 0.001) (Table 2, Fig. 1). We tested whether the giant panda was area-sensitive by testing whether the occupancy probability of giant panda in a patch of a given size was greater than the probability driven only by passive sampling, in which large patches have a higher probability of being occupied at random than smaller patches^46^,^47^,^48^. We used a curve, for the response of occupancy rate to patch area only due to passive sampling, as the null response curve. Occupancy probability to patch area exceeded the null response curve after 40.4 km^2^ (Fig. 1), suggesting area-sensitivity of giant panda.

**Figure 2:**
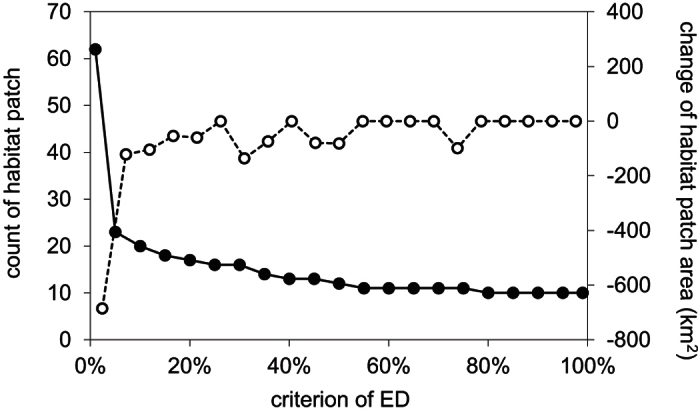
The function *EDp (1%* *≤* *p%* *≤* *99%*) against *p%*, shows the effects of criterion choice on the amount of habitat patches that match the criterion. The solid line with filled circles is the patches count; the dashed line with open circles represents the change in patch area with increasing *p%*.

**Figure 3:**
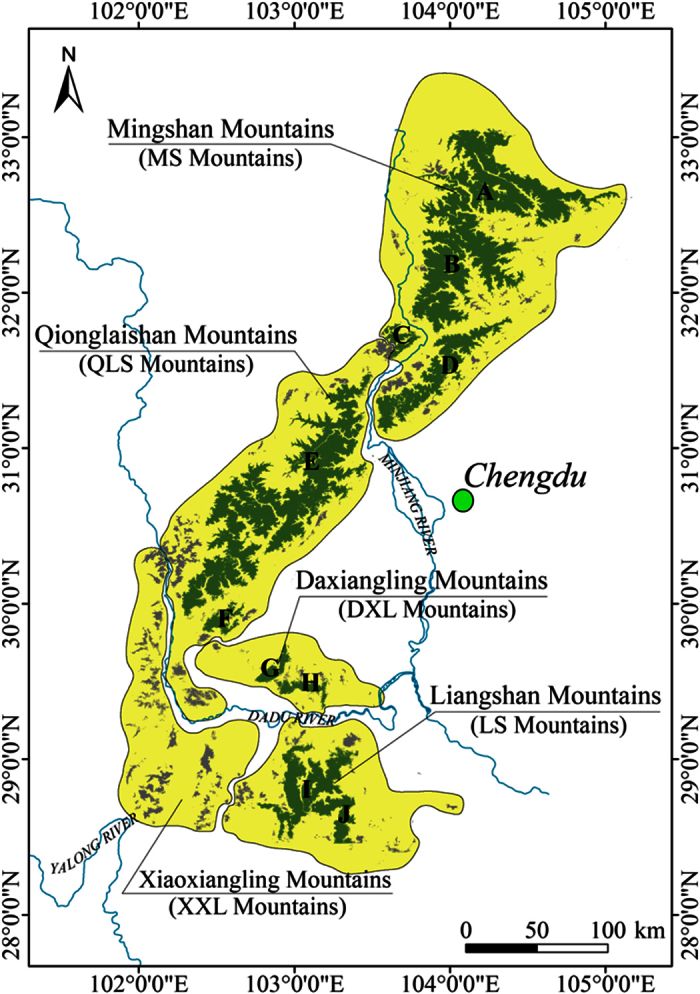
Distribution of habitat patches in the five mountain ranges. Yellow regions show the schematic of the mountain ranges. Habitat patches greater than MAR are green and patches smaller than MAR are grey. The map is made by ArcGIS 9.1 software, http://www.arcgis.com/features.

We calculated the value of *ED*_*p*_, i.e. the patch area at which it is likely that pandas were present with probability of *p*%. Both counts and total sizes of habitat patches with size over *ED*_*p*_ decreased with the increasing of *p*, but they did not change from *ED*_*80*_ to *ED*_*99*_ (Fig. 2). At the 90% effective dose (*ED*_*90*_, indicating the patch area at which it is 90% likely that pandas were present), the value of MAR was estimated as 114.7 km^2^. Ten habitat patches exceeded the estimated value of MAR, which covered 86.1% of the habitat of giant panda. Six habitat patches greater than the MAR were in the MS and QLS Mountains, including the four largest patches. Two patches whose sizes exceed the MAR threshold were located in the LS Mountains, and another two patches in the DXL Mountains just reached the MAR threshold. In contrast, the habitat in the XXL Mountains was highly fragmented, consisting of 1,326 habitat patches, none of which exceeded the MAR threshold (Fig. 3).

## Discussion

Using the latest and most complete empirical data of giant panda presence and land cover, we analyzed the response of occupancy probability of giant panda against habitat patch size. The occupancy probability of giant panda was positively associated with habitat patch areas, and the changes in occupancy as a function of patch area exceeded effects due only to sampling. These results suggest that giant panda is indeed area-sensitive, implying that the size of individual habitat patches is important to giant panda conservation, as is total habitat and population sizes.

Though area-sensitive species are believed to be absent from small habitat fragments, the area-sensitivity of species are mostly identified by the relationship between the patch-specific density of individuals and patch size^49^,^50^,^51^,^52^. For sparsely distributed species, where calculated densities can be strongly influenced by small numbers of individuals, occupancy rather than density is the preferred metric for assessing area-sensitivity. Generally, low probability of occurrence for wildlife in small patches is attributed to demographic stochasticity^53^,^54^, environmental stochasticity and catastrophic events^55^,^56^, inbreeding and loss of heterozygosity^57^, edge effects^58^, food shortages in small patches^15^, and landscape structure^59^. Our study went further, comparing the occurrence-patch size curve with a null response curve, demonstrating that area-sensitivity in giant pandas is real and not just an effect of patch size sampling.

The MAR was estimated to be 114.7 km^2^ using a logistic regression of habitat patch size on giant panda occupancy. MAR can also be estimated from Population Viability Analyses (PVAs)^19^. A MAR of 156–248 km^2^ for giant panda was estimated from its PVA (40 individuals)^60^ and home range size (3.9–6.2 km^2^)^29^. The MAR value estimated from PVA and home range size is higher than that estimated from occupancy patterns and patch area. This discrepancy may arise because giant panda home ranges often overlap^61^,^62^, which will inflate estimates of required space in PVAs. MAR estimated from occupancy patterns are believed to be sensitive to transient dynamics, while MAR estimated using the PVA-based approach enable us to consider time horizon and extinction probability^19^. As a mechanism-driven model, the plausibility of the PVA-based approach is dependent upon the reality of the modeling assumptions and the robustness of model behavior when population and environment parameters cannot be accurately determined, both of which are, often untested. Meanwhile, occupancy data is an empirical synthetic result of all ecological factors and processes in the research area. MAR estimated from occupancy data, together with that estimated by a PVA-based approach, can produce more applicable conservation planning and policy.

Giant panda habitats are large and continuous in the MS and QLS Mountains, preserving approximately 45 and 36% of giant panda habitat in the five mountain ranges, respectively. Residential areas and farmland along roads and valleys segment the habitat into several patches, of which Patch A, B, C and D (in Fig. 3) are large enough to hold relatively viable populations. The gaps between Patch A, B and D are narrow, therefore establishing corridors could further enhance the situation of populations in those patches. It is interesting that giant panda have not been found in patch C at least since the 1980s, even though this patch exceeds the MAR threshold and has a high habitat suitability index. This absence of giant panda may occur because patch C is isolated from other habitat patches in the MS and QLS Mountains by the Minjiang River (Fig. 3), a major tributary, and by human communities along those rivers. Patch E accounted for about 85% of the total area of habitat patches in the QLS Mountains. Several habitat patches, each smaller than the MAR but still containing giant panda presence records, were distributed around Patch E. Those records may stem from ‘spillover’ of giant panda from patch E rather than separate viable populations^63^. Therefore, we urge connection of these small habitat patches to Patch E to enhance the persistence of giant panda in the region.

Though habitats are fragmented in the DXL and LS Mountains^44^, four habitat patches (Patches G-J) exceed the giant panda’s MAR. Establishing corridors to link Patches G and H with other patches in the QLS Mountains could be particularly beneficial for giant panda by creating a single patch substantially larger than the MAR. Such corridors may be possible because there is no major river or other barrier separating the patches in question. In contrast, habitat patches in the LS Mountains have been isolated from adjacent giant panda habitat in the DXL and XXL Mountains due to the Dadu River and a wide stretch of human settlements. As a result, connecting patches in the LS Mountains to those in other mountain ranges appears infeasible. Nevertheless, within the LS Mountains, the gap between Patches I and J is very narrow, suggesting it might be possible to link the patches with corridors.

Of all habitats in the five mountain ranges, those in the XXL Mountains are the most fragmented. No patch in the XXL Mountains exceeds the MAR, and the largest patch therein was a mere 81.7 km^2^. Unsurprisingly, the population of giant panda in the XXL Mountains is the smallest and most endangered of all. To help improve this population, giant panda have been translocated from captivity to the XXL Mountains^64^. However, Veitch^65^ suggested that revealing and repairing the original cause of population degrading is the most important prerequisite to successful translocation. Our MAR analyses imply that these translocation efforts are insufficient to guarantee the long-term survival of local populations, unless efforts are also made to reduce habitat fragmentation. Two parallel efforts would be beneficial. One effort should focus on restoring habitats to increase patch size and suitability for giant pandas. The other should focus on creating ‘corridor groups’^66^ to connect highly fragmented patches inside the XXL Mountains. Together these two restoration strategies would consolidate several small patches into a bigger one, thereby helping to relieve the problem of giant panda habitat fragmentation.

This study has examined only the effects of habitat patch area on giant panda occupancy. Habitat isolation is another key factor that can influence occupancy patterns in other systems^5^,^67^,^68^,^69^. However, we did not explore the effects of isolation on giant panda occupancy here because there was a strong negative correlation between patch size and distance to the nearest patch. Nevertheless, isolation should be considered in future studies of giant panda occupancy that explicitly adopt a more mechanistic perspective on giant panda dispersal.

We determined the value of MAR based on the response of giant panda occurrence probability against habitat patch size. The landscapes and forest habitats in which giant panda live have changed considerably in the five mountain ranges over the last few decades, and the impacts of these changes on populations may lag behind the physical changes themselves. For example, because wild giant pandas may live as long as 13.3 years^70^, a shrinking habitat patch can still be occupied by giant pandas for many years beyond when the patch size falls below the true MAR, due to extinction debt^71^ or just because of the stochastic nature of extinction^72^,^73^. This time lag effect introduces a downward bias on the estimated MAR because the occurrence patterns have not equilibrated to habitat changes. In contrast, though rare, landscapes where dispersal is sufficient to maintain long-term connections between nearby patches (e.g., landscapes where big river gorges, farming areas, communities and roads introduce transient but not permanent barriers to connectivity; for example, Patch C) would bias the estimated MAR upwards. Because time lags, rare dispersal events, and other factors will influence giant panda occupancy patterns over the long-term, our calculated MAR values are merely estimates of the relationship between giant panda occupancy and patch size. Nevertheless, these estimates provide helpful guides to assist in identifying priority areas for restoration efforts.

We identified habitat patches using HSI, but this does not negate the importance of areas outside the patches for the viability of giant panda. Almpanidou *et al.*^74^ suggest that conservation planning should consider the entire habitat rather than isolated patches of high quality, because less suitable areas can be indispensable connections among habitat patches. That connection is essential for the viability of populations, especially those in small patches^75^. Future work should analyze habitat connectivity and plan corridors to enhance the viability of giant panda populations^76^, particularly in southern mountain ranges where most habitat patches are smaller than the MAR. Because fragmentation of habitats may be aggravated in the future due to possible global climate change^76^,^77^,^78^, it is necessary to evaluate their status within that context and investigate potential solutions.

## Methods

### Data and data sources

All of the presence records (feces, footmark and forage traces) for giant panda in the five mountain ranges (i.e. Minshan Mountains, Qionglaishan Mountains, Daxiangling Mountains, Xiaoxiangling Mountains and Liangshan Mountains) were obtained from the Fourth National Giant Panda Survey (NGPS4) in Sichuan province. The survey was carried out between 28°10′ N and 33°48′ N, 101°50′ E and 105°28′ E, from 2011 to 2013, and covered about 330,000 km^2^.

Giant panda habitat patches were identified by 29 spatially explicit environmental variables and classified into two categories, namely geography and land use (Supplementary Information). Elevation data were obtained from the Digital Elevation Model (DEM) (30 m × 30 m), provided by the International Scientific & Technical Data Mirror Site, Computer Network Information Center, Chinese Academy of Sciences (http://www.gscloud.cn). Other geographic variables (slope, curvature, topography position index (TPI), aspect, solar radiation index, and latitude) were derived from the DEM. Information on land use was obtained from the Second National Forest Inventory (NFI2) and revised by the NGPS4 dataset.

### Habitat patch identification

The habitat suitability index was evaluated using MaxEnt modeling^79^,^80^. This technique, which has its origins in statistical mechanics^81^, builds a map of a species’ likelihood distribution by estimating the probability distribution of maximum entropy^82^,^83^. The presence records of giant pandas were thinned randomly to consolidate presence records from sites separated by less than 1,125 m. The distance of 1,125 m was roughly determined by the value of the smallest home range of giant pandas (3.9 km^2^)^29^. Overall, 4,224 records of giant panda presence were consolidated to 1,421 valid presence sites.

We did not try to reduce the collinearity by eliminating strongly correlated environmental variables, because high collinearity is not a significant problem for machine learning methods if the goal is predicting presence rather than interpreting the response of presence to environmental variables^84^. Moreover, eliminating variables may discard critical information for prediction, even when those variables are strongly correlated with other variables. For example, the variable of slope is strongly correlated with altitude (*r* = 0.8469, *P* < 0.01) at the scale of the five mountain ranges, whereas slope is apparently indispensable for predicting giant panda presences at finer scales.

We measured model performance using the area under the receiver-operator curve (AUC)^85^ and the True Skill Statistic (TSS)^86^. A value of AUC greater than 0.90 was considered to be excellent, 0.8 to 0.9 good, 0.7 to 0.8 fair, and 0.6 to 0.7 poor^87^. For TSS values, greater than 0.75 was considered to be excellent, 0.4 to 0.75 good, and less than 0.4 poor^88^.

Using the reduced set of valid presence recodes and environmental variables, we performed a 10-fold cross-validation procedure to create MaxEnt models. The averaged AUC and TSS across the 10-fold test sets were calculated to determine the predictive power of the models. We delineated the averaged HSI into suitable and unsuitable habitat based on the threshold value that would maximize the TSS score, i.e., the sensitivity-specificity sum maximization approach^89^,^90^. There are many approaches to determining the thresholds to transform results of species distribution modeling from suitability for species occurrence to presences/absences. Liu *et al.*^91^ compared 12 methods and suggested that sensitivity-specificity sum maximization, which we adopted, was a good approach.

Those adjacent suitable habitat cells were joined together as a habitat patch following an 8-cell rule. The 8-cell rule considers all 8 adjacent cells, including the 4 orthogonal and 4 diagonal neighbors. A habitat patch is considered occupied if any presence record of giant panda (valid or not) is located in it.

### Area-sensitivity identification

Identifying whether a species is area-sensitive is tricky. An occupancy rate increasing with patch size does not necessarily indicate area-sensitivity because occupancy of a species may be higher in bigger habitat patches than in smaller ones due to passive sampling. Specifically, if a patch has an area of *a*, and the occupancy rate of a species in that patch is *q*, then in a bigger patch with an area of *na*, the occupancy rate will be 1 − (1 − *q*)^*n*^ in the absence of any area-related ecological effects. In contrast, a species that is truly area-sensitive should have an occupancy curve that increases faster than that of passive sampling as patch size increases. A variety of mechanisms contribute to this effect. For example, bigger patches can increase the availability of ‘core’ habitat area unaffected by edge effects or human disturbance, and thus are favorable for species that require interior habitat^92^. Therefore, an area-sensitive species should show a higher occupancy rate than that driven by passive sampling alone.

To test whether the occupancy rate of giant panda in patches of given size is higher than that driven by passive sampling, we compared the observed logistic occupancy curve with a null response curve. The null response curve is defined by the function 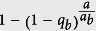, where *a*_*b*_ (corresponding to the minimum home range size of a giant panda^29^, namely 3.9 km^2^) and *a (a* > *a*_*b*_) is the patch size, *q*_*b*_ is the occupancy rate in a patch with size of *a*_*b*._ The null response curve corresponds to the probability of a habitat patch of size *a* being occupied by giant panda, provided that patch is ecologically and functionally equivalent to 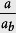 independent patches with areas of *a*_*b*_ and shows no area-related ecological advantages over the set of small patches.

### Estimating MAR

We progressed from occurrence estimates to estimates of giant panda’s area needs by leveraging dose-response curves from pharmacology^93^,^94^ in which the key measure is the effective dose (ED) that yields a particular probability. Thus, in the present study, *ED*_*p*_is the patch area at which it is likely that pandas were present with probability of *p*%. We used the *ED*_*90*_ as the value of MAR. The *ED*_*90*_ was derived from a logistic regression of probability of occurrence against patch size. The interpretation of *ED*_*90*_ is that 90% of patches of that size will support pandas. The *ED*_*90*_ is sometimes used as an estimation of the maximal effective dose in drug development^95^. Though we used a threshold of 90% to determine MAR, the choice of threshold may vary among conservation programs depending on specific objectives. Therefore, we plotted the function *ED*_*p*_ (*1*% ≤ *p%* ≤ *99*%) against *p%*, to show the effects of criterion choice on the amount of habitat patches matching the criteria.

## Additional Information

**How to cite this article**: Qing, J. *et al.* The minimum area requirements (MAR) for giant panda: an empirical study. *Sci. Rep.* **6**, 37715; doi: 10.1038/srep37715 (2016).

**Publisher's note:** Springer Nature remains neutral with regard to jurisdictional claims in published maps and institutional affiliations.

## Supplementary Material

Supplementary Information

## Figures and Tables

**Table 1:** Count and area of giant panda habitat patches in five mountain ranges.

Mountain ranges	Number of habitat patches	Habitat patch area (km^2^)	Area of mountain ranges (km^2^)
MS	1262	6845.5	48438.9
QLS	1389	5561.6	27841.5
DXL	542	422.9	8136.7
XXL	1326	748.6	26877.1
LS	1050	1895.6	15828.5

The habitat suitability index was evaluated using Maximum Entropy (MaxEnt) modeling. Habitat patches were identified using HSI and a threshold value that maximized the TSS score.

MS: Minshan Mountains; QLS: Qionglaishan Mountains; DXL: Daxiangling Mountains; XXL: Xiaoxiangling Mountains; LS: Liangshan Mountains.

**Table 2:** Logistic regression model of occupancy probability of giant panda against habitat patch size.

Variable	Coefficient	SE	*Z*	*P* > *|z|*
Occupancy probability against patch size				
Constant	−5.194	0.180	−28.837	<0.001
Area	0.0644	0.0099	6.459	<0.001

SE: Standard error.
