# Supplementary material for: The minimum area requirements (MAR) for giant panda: an empirical study
Source: Sci Rep. 2016 Dec 8;6:37715. doi: 10.1038/srep37715 (PMC5144585; doi:10.1038/srep37715)
Supplement: Supplementary Information [file srep37715-s1.pdf]

Manuscript number: SREP-15-21272

Manuscript title: the minimum area requirements (MAR) for giant panda: an empirical study

Authors: Jing Qing, Zhisong Yang, Ke He, Zejun Zhang, Xiaodong Gu, Xuyu Yang, Wen Zhang, Biao Yang, Dunwu Qi , and Qiang Dai

Environmental variables used for identify panda habitat patches

**Class**	**Variable (abbreviation)**	**Source**
geography	Elevation (DEM)	Scientific database of the Chinese Academy of Science
	Slope	Derived from DEM
	Curvature	Derived from DEM
	Topography position index(TPI)	Derived from DEM
	Aspect	Derived from DEM
	Solar radiation index	Derived from DEM
	Latitude	Derived from DEM
land use	The distance from meadow	NFI2*
	The distance from evergreen deciduous broad-leaved mixed forest	NFI2
	The distance from evergreen shrub-land	NFI2
	The distance from evergreen broad-leaved forest	NFI2

The distance from shrub-grassland	NFI2
The distance from cold-temperate coniferous forest	NFI2
The distance from deciduous shrub	NFI2
The distance from deciduous broad-leaved forest	NFI2
The distance from warm coniferous forest	NFI2
The distance from temperate conifer and broad-leaved mixed forest	NFI2
The distance from temperate coniferous forest	NFI2
The distance from sclerophyllous evergreen broad-leaf forests	NFI2
The distance from cultivated vegetation	NFI2
The distance from cultivated bamboo	NFI2
The distance from cultivated crops	NFI2
The distance from bamboo	NFI2
The distance from water body	NFI2
The distance from developed land	NFI2
The distance from freeway	NFI2
The distance from national road	NFI2
The distance from provincial highway	NFI2
The distance from county road	NFI2

* NFI2: The Second National Forest Inventory
